# Spatial Aspects of Health—Developing a Conceptual Framework

**DOI:** 10.3390/ijerph20031817

**Published:** 2023-01-19

**Authors:** Jobst Augustin, Valerie Andrees, David Walsh, Ralf Reintjes, Daniela Koller

**Affiliations:** 1 Institute for Health Services Research in Dermatology and Nursing (IVDP), University Medical Center Hamburg-Eppendorf (UKE), 20246 Hamburg, Germany; 2 Glasgow Centre for Population Health, Glasgow G40 2QH, UK; 3 Department of Health Sciences, Faculty of Life Sciences, Hamburg University of Applied Sciences, 20999 Hamburg, Germany; 4 Health Sciences Unit, Faculty of Social Sciences, Tampere University, 33100 Tampere, Finland; 5 IBE—Institute for Medical Information Processing, Biometry and Epidemiology, Ludwig-Maximilians-Universität München, 81377 Munich, Germany

**Keywords:** health, space, determinants, geography, conceptual framework

## Abstract

Numerous studies and models address the determinants of health. However, in existing models, the spatial aspects of the determinants are not or only marginally taken into account and a theoretical discussion of the association between space and the determinants of health is missing. The aim of this paper is to generate a framework that can be used to place the determinants of health in a spatial context. A screening of the current literature first serves to identify the relevant determinants and describes the current state of knowledge. In addition, spatial scales that are important for the spatial consideration of health were developed and discussed. Based on these two steps, the conceptual framework on the spatial determinants of health was derived and subsequently discussed. The results show a variety of determinants that are associated with health from a spatial point of view. The overarching categories are global driving forces, policy and governance, living and physical environment, socio-demographic and economic conditions, healthcare services and cultural and working conditions. Three spatial scales (macro, meso and micro) are further subdivided into six levels, such as global (e.g., continents), regional (e.g., council areas) or neighbourhood (e.g., communities). The combination of the determinants and spatial scales are presented within a conceptual framework as a result of this work. Operating mechanisms and pathways between the spatial levels were added schematically. This is the first conceptual framework that links the determinants of health with the spatial perspective. It can form the working basis for future analyses in which spatial aspects of health are taken into account.

## 1. Introduction

Health status is shaped by a multitude of individual characteristics: Behaviours, socioeconomic background, age, sex/gender, cultural background, educational attainment, genetics, and of course, the interaction of all those factors. However, individuals exist in a geographical setting—in our households with family, in neighbourhoods, regions, countries, continents—and each geographical level encompasses more people, cultures, public policies, environmental and infrastructural factors that might also play a role in shaping that person’s health. The combination of individual characteristics and the surrounding influences which interact and inform each other is the highly complex topic of the framework proposed within this paper. It leans on the scientific fields of medical and health geography which have long historical roots. The interaction between place and health has been of interest for a very long time, starting with the Greek physician Hippocrates of Kos (460–370 BC). He formulated the association between geography and health in ancient times when he attributed disease to the influences of climate, soil, water, lifestyle and nutrition [1]. Following Hippocrates, the miasma theory was the prevailing paradigm of the origin of diseases. The theory is based on the idea that toxic conditions that are inhaled cause diseases such as malaria or cholera [2]. Over the course of time, the importance of geographical aspects of health have been repeatedly shown. For example, John Graunt was the first to investigate the relationship between infant mortality and its seasonal variation in the 17th century [2]; he also calculated the first mortality tables as part of his endeavours [3]. Another example is Sir Percival Pott (1714–1788) who established the association between environmental factors and cancer by showing that chimney sweeps were more likely than other occupational groups to develop testicular cancer, and he theorised that contact with soot could be a cause. The modern era of medical geography originates in the 19th century, when maps of disease incidence were created to describe the spread and possible causes of outbreaks of infectious diseases such as yellow fever and cholera [4]. The most well-known and cited example in the field of public health and place is certainly John Snow with his map of the cholera epidemic in London, even if his contribution of a map-based public health intervention (the removal of the pump on Broad Street after identifying it as the cause of the outbreak) might be a scientific myth [5].

Since then, the thematic focus, as well as the applied methods, have expanded massively. The field of medical geography, focusing mostly on disease ecology, has evolved towards a more societal perspective and of course took advantage of new methodological possibilities, data sources and information from neighbouring disciplines (e.g., statistics, computer sciences, social sciences) [6,7]. The perspective on health (instead of just disease) has also become increasingly prevalent. This was also accompanied by the designation of the discipline from “Medical geography” to “Health geography”.

Not only has the field of Health geography grown, but the consideration of spatial aspects of health has also made its way into other scientific disciplines (e.g., public health, epidemiology, health economics). These spatial perspectives include the whole population perspective and examine the variation in healthcare provision and utilisation, utilise qualitative methods, quantitative analyses and geographic information system (GIS) methods. This broadening of the field also comes with a wide interdisciplinarity of scientists focusing their work on the geographical aspects of health: Physicians, geographers, social scientists, epidemiologists and public health experts, but also non-scientific institutions of healthcare such as ministries or health insurers use regional health data to show spatial variations, identify areas of need for targeted interventions and to identify over- or underprovided regions.

Such interdisciplinarity is a great advantage for researching and comparing different populations living in different environments: Their (health) needs, expectations and (socioeconomic, genetic, demographic) background factors. These environments can differ e.g., in access to green space, noise or air pollution, in neighbourhoods of high or low socioeconomic standing. The interactions of place and health can happen at different levels. For example, on a global level, the impact of climate change, or the role of international travel on the spread of diseases. The national scale could include the health system infrastructure of where physicians or pharmacies are located or how health providers are trained. At the local/community level, one focus might be whether healthy food is available or what local physical activity programmes are in place. At the neighbourhood level, there may be interest in whether there are accessible green spaces or public transport. All those geographical levels and thematic focuses also depend on who lives where, the socioeconomic and demographic composition of the areas as well as peoples’ health needs.

While very practice-orientated, the discipline is still lacking in theory. Health geography has often borrowed frameworks from various disciplines that are applicable to this context. In addition, however, there are also theoretical ideas, for example on the influences of the neighbourhood [8,9,10,11,12]. The places in which people live and work have an enormous impact on their health, and the importance of place to health status therefore became increasingly clear in the last decades of the 20th century [13]. It is all the more remarkable that place and its spatial dimensions are not explicitly taken into account in models of health determinants, and that a general framework on the interactions between place and health on multiple layers is still missing. While there are numerous models [14] on the (social) determinants of health [15,16], they do not explicitly consider the different spatial levels. This paper illustrates why this is needed and suggests a framework of spatial determinants of health. Since a globally applicable and universally valid framework is hardly feasible, the focus in this paper is on high-income countries.

The paper first describes the influencing factors on a person’s health and their potential interactions. Of course, the individuals’ perspectives, background and attitudes play a major role in this. We then describe the process as to how we chose meaningful geographical levels. Through the synthesis of the findings, a conceptional framework is proposed and discussed.

## 2. Determinants of Health on Different Spatial Scales

The selection of health determinants presented here are primarily those highlighted in the literature; we then consider them explicitly within the spatial context. We describe these determinants and spatial attributes individually; clearly, however, in reality they cannot be viewed in isolation, but rather as factors that influence each other.

### 2.1. Global Driving Forces

Over the last decades, global health issues have become more prominent. Examples include events such as the HIV/AIDS pandemic, severe acute respiratory syndrome (SARS) outbreak, Ebola virus in West Africa, Zika virus in the Americas or the current COVID-19 pandemic (SARS-CoV-2) [17]. However, it is not only infectious diseases that are of global importance. Non-communicable diseases such as diabetes [18], cancer [19], obesity [20] and cardiovascular diseases [21] are becoming more important globally, at least in high income countries. However, the literature shows that countries that are not classically high-income countries are also increasingly affected [22]. Depending on the region (high- and low-income countries), the occurrence and causes of these diseases might be very different. What they have in common, however, is that changes at the global level are of great importance. Global driving forces are not the sole cause of this development, but they are of overriding importance.

To date, there is no known uniform definition of global driving forces that affect health. Rather, they must be seen in the context of the respective disease. For example, Austvoll et al. describe that global changes caused by urbanisation, loss of biodiversity, industrialisation and land use are partly responsible for a dysbiosis of the microbiota and increasing prevalence of obesity [23]. In addition, SARS-CoV-2 is a good example for global changes and the emergence and spread of a disease. Barouki et al. and Calistri et al. identified the association to urbanisation, habitat destruction (loss of biodiversity), land use changes and global travel for the global spread of SARS-CoV-2 [24,25]. There is also considerable discussion about the extent to which climate change has contributed to the emergence and spread of COVID-19 [24,26].

The connections between global changes and consequences for health are often multifactorial and very complex. Regardless of this, it is important to consider global, i.e., mesoscale changes, and their consequences for health. Thus, the United Nations (UN) General Assembly also emphasises that governments need to pay more attention to global health in their foreign policies and strengthen their negotiations and political interactions in this area [27].

### 2.2. Policy and Governance

There is a wealth of evidence regarding the impact of political and economic factors (often termed the ‘political economy’ [28]) on population-level health. This clearly overlaps with the fundamental causes of health inequalities being socioeconomic [29], as is discussed further below; importantly, however, socioeconomic conditions are shaped to a huge degree by political and economic decision-making at different levels of governance, and through different spheres of influence. Examples abound, from 19th century analyses of the causes of adverse living conditions and poor health in different parts of Europe [30,31,32], the extreme effects of political and economic change in 20th century Russia [33], variations in regional [34] and urban [35] health in Europe. Beside this—most recently—hugely concerning changes in mortality rates associated with government austerity (cuts to public spending) policies in many high-income countries [36], and most notably in the UK [37,38]. A recent systematic review of reviews of the impact of the political economy on population health demonstrated positive health impacts from social democratic welfare states, higher public spending, fair trade policies, extensions to compulsory education provision, microfinance initiatives in low-income countries, health and safety policy, improved access to health care and high-quality affordable housing, while in contrast, negative impacts in terms of wider income and health inequalities, worse self-rated health and higher mortality were associated with more neoliberal policy approaches (the latter defined as the advocacy of individualism, marketisation and privatisation of industry, goods and services, and the financialisation of large sections of the economy) [28]. Perhaps the most famous quote associated with understanding the political solutions to poor health outcomes in societies is that of the German physician Rudolf Virchow, who stated in the 19th century that “medicine is a social science, and politics is nothing else but medicine on a large scale” [32].

### 2.3. Sociodemographic and -Economic Conditions

As stated above, there is a clear overlap between the political economy and the broader socioeconomic conditions that are prevalent in society; importantly, the former strongly influences the latter. However, the importance of such societal conditions for health cannot be over-emphasised. There is an enormous array of evidence, spanning both time (centuries rather than just decades) and place, which has demonstrated the clear relationship between income, poverty, socioeconomic position (alongside other factors including educational attainment) and an equally broad set of different health outcomes and determinants [39,40]. As a consequence, there is a clear social gradient in health across societies, with each step higher up the socioeconomic ladder associated with better health [41]. Thus, as already stated, the fundamental causes of health inequalities are socioeconomic, and importantly, these endure over time. As Link and colleagues have stated, “a broad range of circumstances that affect health are shaped by socioeconomic resources… [and these were] equally as useful in avoiding the worst sanitation, housing, and industrial conditions of the 19th century as they are in shaping access to the current circumstances” [29].

### 2.4. Living Environment

The living environment has a high significance for health, as many studies show [42]. Tiwari et al. defined the living environment as an “assembly of the natural and built environment which is offered to the inhabitants of the place who perform various kinds of social, cultural, religious, economic, and political activities which induce peculiarities in the character of the living environment” [43]. According to this definition, it can be seen that the living environment is a very broad concept including the individual’s home and direct surroundings, but also their workplaces, places they shop, use of services or socialise, and includes many aspects that influence health.

Air pollution, as part of the local living environment, is not only the cause of health problems in so-called emerging countries. In high income countries, too, specific respiratory diseases (e.g., chronic obstructive pulmonary disease (COPD), bronchial asthma, bronchitis) can be traced back to polluted air (e.g., road traffic) [44]. Not only the respiratory tract is affected by air pollutants but also the cardiovascular system [45] and other organs such as the brain. Noise, especially road traffic and aircraft noise, is considered to be a relevant risk factor for adverse health effects [46]. Several studies have shown the link between noise and different cardiovascular and mental health outcomes [47]. It is important to point out that air pollution and noise often occur together (e.g., from road traffic) and thus have a particularly strong impact on health.

In addition to the above-mentioned factors that can be attributed to the physical environment, green [42] and blue [48] spaces have an important function for health in the living environment. For example, Maas et al. investigated the relationship between the proportion of urban green space in the residential environment and the morbidity of residents on the basis of 24 selected non-communicable diseases (e.g., high blood pressure, osteoporosis, depression, asthma, diabetes) [49]. It was found that the prevalence of 15 of the 24 diseases (e.g., depression, anxiety disorder) was in part significantly lower, if there was a high proportion of urban greenery close to home (<1 km).

The willingness to engage in physical activity (e.g., walkability, bikeability) is closely associated with the availability of green and blue spaces. It has long-term benefits for preventing chronic diseases and improving mental health problems and quality of life (QoL) [50]. The results of a systematic review [51] showed significant increased effects of green and blue physical activity interventions on physical and mental health.

A healthy diet is important for the prevention of diseases (e.g., hypertension, diabetes). In recent decades, numerous studies have examined the relationship between nutrition, the living environment and access to healthy and nutritious food. Particularly in the English-speaking research community, various concepts have emerged around nutrition and access to healthy nutrition and healthy living environments. These include the concepts of food deserts [52], foodscapes [53] or the obesogenic environment [54]. The studies show that the structure and distribution of food retailing are spatial indicators of social structure. This is reflected in unequal access opportunities, nutritional behaviour and health status depending on social status [55].

### 2.5. Healthcare Services

There are considerable regional variations in both the provision and the utilisation of health services. On the one hand, these differences may be planned and even intentional, as people with different healthcare needs live in different places and specialist care will likely be concentrated in bigger hospitals or urban areas. According to Wennberg, these regional variations caused by the composition of the population are an expression of the right care. On the other hand, variations can be problematic if they are generated by the providers or by patient preferences or provider factors, leading to a lower quality or more expensive care [11]. A main focus of the geographical research in healthcare services is understanding if and why variations are valid, and what consequences derive from unwarranted variations caused by underuse, overuse or misuse.

An important aspect related to regional variations in provision and utilisation of care is the geographical aspects of healthcare accessibility. The concept of accessibility is multidimensional, with the most commonly cited five dimensions being availability, (geographic) accessibility, accommodation (organisational structure), affordability and acceptability [56]. This model has been adapted to other contexts, e.g., for low- and middle-income countries (LMIC) [57]. In more recent publications, awareness (communication and information from providers to patients) is included as an additional dimension [58].

### 2.6. Cultural and Working Conditions

People’s health is closely related to everyday routines and traditions, which in turn are strongly influenced by culture. Dietary and consumption behaviour (smoking, alcohol) serve as good examples. Our diet is, besides other influences (e.g., affordability), essentially influenced by our culture. Eating habits change along with lifestyles and under the influence of social developments. Besides this, increased globalisation and the emergence of multicultural communities has led to further changes in dietary habits in Europe [59]. While fast food is still popular, especially among young people, trends towards a health-conscious and sustainable eating culture can also be observed in the 21st century, also including vegetarian or vegan diets. Cultural norms and beliefs are also strong predictors of alcohol consumption [60]. Regardless of race and ethnicity, African Americans and Latinos have more conservative attitudes towards drinking compared to whites [61], which translates into lower alcohol consumption. Cultural norms also vary by context and place. Ahern et al. found that neighbourhood norms against drunkenness were a more robust and stronger predictor of binge drinking than permissive beliefs about it held either by the individual or family and friends [62,63]. Cultural norms are not only important for the consumption of alcohol, but also for tobacco consumption. Cultural values and social beliefs are important factors that affect smoking status and determine whether smokers continue this behaviour or successfully quit [64]. Cultural background is not only important for risk behaviour but also preventive behaviour, e.g., cancer screening. In the US, differences in participation in colorectal cancer screening between different ethnic minority groups and non-Hispanic white populations can be attributed to cultural differences. Attitudes and beliefs towards cancer and screening—such as fear, anxiety and fatalism—are important and strong predictors of low rates of colorectal cancer screening [65,66,67].

Work has an important influence on the daily routine and a significant part of the day is spent at work. Accordingly, work itself, but also the work environment, has a high importance for physical and mental health. Work stress and unemployment have long been recognised as social determinants of health and a number of job demands, job characteristics and occupational stresses are associated with socioeconomic or occupational status. Thus, the work environment is also considered one of the origins of social inequalities in health [39,68,69,70]. For example, studies show that physical working conditions (e.g., contact with toxic dusts, vapours) have a clear social gradient and clearly mediate the relationship between social status and health. In contrast, the effects of psychological work demands or psychosocial work factors have so far been less clearly linked to social status and health [70,71,72,73].

**Figure 1:**
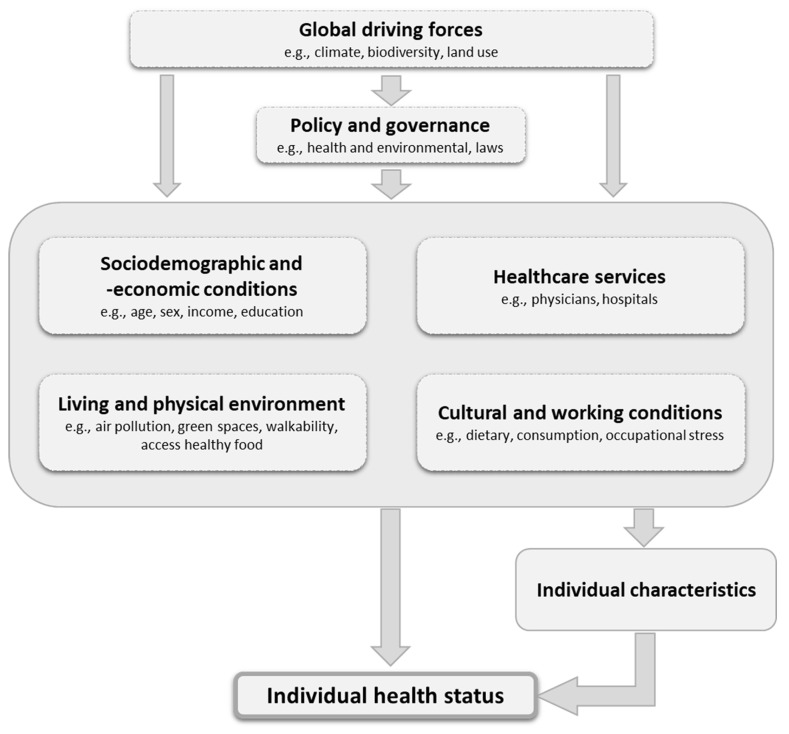
Determinants of health.

Figure 1 presents an overview of the determinants of health described above and their association with individual health. Sociodemographic and economic conditions, health services, living and physical environments and cultural and working conditions are boxed together because these determinants influence each other. In addition, they are controlled by global forces as well as politics and governance. They also directly influence individual health status as well as individual characteristics (e.g., genetics, lifestyle, and behaviour); in turn, the latter exert influence on individual health status.

## 3. Conceptual Framework: Spatial Determinants of Health

One of the key issues in developing the conceptional framework is the choice of geographical levels. Based on published papers, we identified theorisation of different spatial levels: Rodrigue et al. distinguish between city (macro level), district (meso level) and community (micro level) in the spatial organisation of transportation systems [74]. Sternlieb and Laituri have developed a concept of nested spatial hierarchy and water resource activity that distinguishes between five levels: Global scale, continental scale, national scale, basin scale and community scale [75]. Amin also makes a very differentiated distinction in the urban Strategy for Sustainable Urbanisation and distinguishes between global, region, country, metropolitan, community, neighbourhood and household level [76]. It can be seen that the differentiation of spatial levels is primarily in the context of the topic and that there is no generally applicable concept for spatial scaling.

With regard to health geography, no comprehensive concepts or frameworks exist that are based on the hierarchy of spatial levels. Maede and Emch use a spatial hierarchy to visualise health in the neighbourhood [77], but it is rather simple. This places neighbourhoods after region and community on one side and above the individual on the other. In other conceptual models, the spatial aspect is mentioned, but is not the focus of analysis [78] or the spatial scale is present for the specific framework (such as for urban health or rural health) [79].

**Figure 2:**
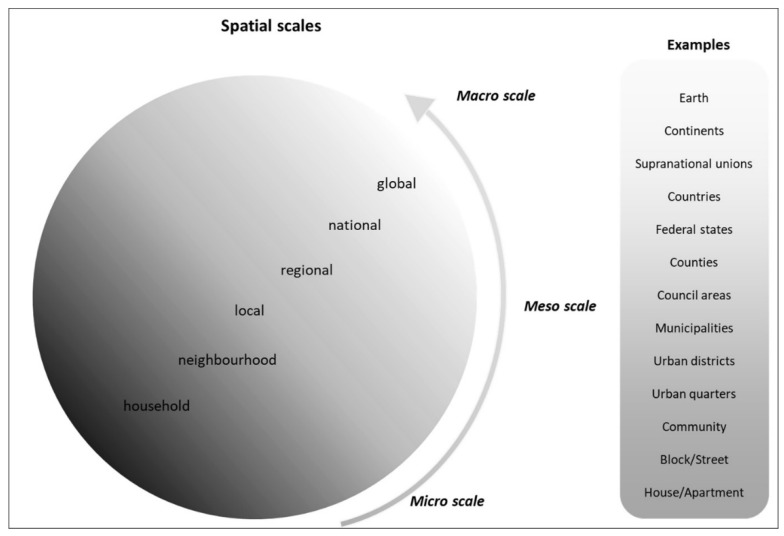
Spatial scales in health.

As the discussion of determinants and their effects has shown, a rather fine differentiation of spatial scales is necessary in the context of health. Due to this, we distinguish between the following six spatial levels: global, national, regional, local, neighbourhood and household. For illustration purposes, the levels are shown with examples in Figure 2.

It should also be noted that although the levels can be distinctly named, the transitions often merge as a continuum. For example, there is no fixed spatial demarcation between neighbourhood level and local level.

**Figure 3:**
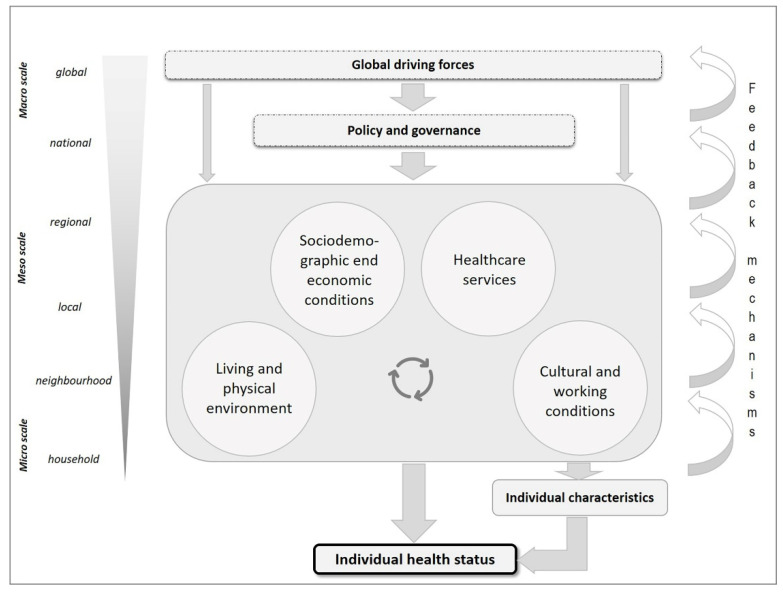
Spatial aspects of health—developing a conceptual framework.

Based on the determinants of health (Figure 1) and the spatial scales (Figure 2), Figure 3 presents the proposed framework. As mentioned above, individual health status is influenced by background (sociodemographic, economic or educational, lifestyle, etc.), but also by a range of external factors. At the global level, the forces of globalisation (e.g., urbanisation, migration), climate change or biodiversity have an impact. They both influence political decision-making (e.g., programmes for climate protection and adaptation at various levels), but also impact directly on individuals’ health. However, national governments not only influence global forces (e.g., by means of international agreements), but also naturally affect the determinants of health within a country through various instruments and legislation (e.g., governance of the health and social system). Both the global driving forces, but especially the political and administrative ones, have an impact on the smaller levels (regional, local, neighbourhood and household): examples include the control of local healthcare supply (e.g., number of hospitals, doctors), and the provision of public transport. The setting and implementation of regulations on air and noise pollution at the place of residence and work can also be assigned to this level. The determinants mentioned in the concept are to be understood as superordinate categories that are interrelated. For example, there is a close association between sociodemographic conditions, the living environment and the availability of healthcare services. These individual characteristics are also influenced by the determinants, at least in part (e.g., behaviour and cultural conditions).

Pathways exist not only from the macro (global) to the micro level (household), but also via feedback mechanisms that exist and work in opposite directions. This means that conditions at a smaller scale can also lead to changes at spatially higher levels. For example, high local air pollution leads to policy measures at various levels, which in turn are intended to improve local air quality. This in turn improves the quality of the living environment and benefits people’s individual health.

## 4. Consequences for Public Health

According to the World Health Organization (WHO), public health has several important tasks, including: health promotion, social mobilisation as well as advocacy and communication for health, surveillance and monitoring of health and hazards, disease prevention (and early detection), health protection, assuring governance for health and wellbeing and improving sustainable structures [80]. All these tasks and activities comprise different spatial levels as illustrated in the conceptual framework. The different spatial scales highlighted in Figure 2 and Figure 3 obviously focus attention on the need for a range of public health responses and actions to be undertaken at those different levels. Of course, not all public health questions need attention at all levels at all times. In the following paragraphs, we give examples of the spatial attributes for the tasks where the spatial levels play an important role.

Health promotion activities are often viewed as having a particular emphasis on individuals, and the choices of individuals. However, the majority of the main health promotion strategies that were set out in the WHO’s Ottawa Charter [81] have clear links to the different spatial scales discussed here. For example, the need to create supportive environments (applicable to multiple geographical layers in Figure 3), strengthening community action (particularly relevant to local and neighbourhood levels) and reorienting health services (at the local, regional and national level). Similarly, the building of healthy public policy (discussed further below) needs to be done across several spatial layers of governance.

The social determinants of health are a key component of Figure 3, and health inequity is a related key outcome. It is well understood that actions to address both are required at the same multiple levels; for example, actions on maximising individual income through both employment earnings and protective social security, the provision of warm and affordable housing, the creation of safe neighbourhoods, other broader environmental measures, education strategies etc.—these all have clear and relevant influences at different spatial scales.

The same is true of the need for public health advocacy and communication at different levels—albeit that it is also important that key public health messages are not communicated in a way that potentially widen—rather than narrow—inequalities [82]. Finally, the importance of governance—applied particularly to political leadership and the broad political determinants of health—has already been emphasised in this paper: Therefore, there is a clear, evidenced need for the implementation of policies to improve wellbeing (e.g., the WHO’s health in all policies approach [83]) and narrow societal inequalities [82,84] to occur at different levels of such governance.

A key task of public health activities in countries or regions is the surveillance of diseases and health status. Surveillance is a term that is used in various settings. In public health and epidemiology, surveillance can be defined as a systematic and dynamic process for the collection, management, analysis, aggregation and reporting of data on the occurrence of incidents (often diseases) in a selected population group. It can be used for early detection of changes in disease frequencies and thus can provide early warning signals [85]. In this way, epidemiological surveillance can play an important part in identifying and comparing politically relevant health issues to guide experts and decision makers [86]. The main criteria which are used to analyse epidemiologic surveillance data are ‘time’, ‘person’ and ‘place’. Time refers to the principle of time series analysis to identify time trends (e.g., the discovery of upcoming outbreaks). Analysing data on the affected populations or individuals gives a better understanding of people at risk, and geographical data are extremely useful in describing the spread of diseases or risk factors. In this area, qualitative research and mixed-methods studies has become more and more important to add to embrace the different perceptions of space and health [87,88]. Over recent years this aspect has become more relevant in the surveillance of population health and well-being and the integration of geographic information systems (GIS) in many surveillance systems has become standard. In particular, the use of interactive maps has proven to be a useful way of communicating trends and developments. If epidemiological data and information are to play a role in policy-making, they need to address issues that are relevant to decision-makers and decision-making processes. However, particular care must be taken to ensure that forms of representation are used that are easy to understand and are of use for the intended audiences. Here, the use of geographical components such as GIS can play an important role in making connections clear [89]. The use of GIS and therefore the incorporation of a spatial perspective in epidemiological surveillance will continue to increase in the coming years and will become a standard tool for research and practice for monitoring and responding to health hazards and emergencies [90], also including the integration of qualitative data [91].

Alongside the above, another fundamental public health task is health protection. This can range from ensuring environmental safety or access to healthy food to offering disease prevention through medical services (e.g., vaccinations) and non-medical services (e.g., the possibility to be physical active in one’s own neighbourhood). Those examples alone already show the need for the spatial perspective: Whereas access to green space, regulation of air pollution, or disease monitoring or vaccinations have an obvious local focus [92,93,94], the broader policies, medical planning and reaction to need are based more on a national or global level [80]. For those, public health and health system responsiveness go hand in hand and in an optimal setting the actions are taken at the spatial levels necessary to ensure the best possible health outcomes for the whole population.

According to the WHO framework for health systems, one of the main tasks of a health system is to ensure the provision of health services, including training the public health workforce, and to develop sustainable (financing) structures [95]. While the health system itself is most often organised at the national level, the implementation of those structures occurs at all geographical levels below this.

## 5. Conclusions

The aim of this article was to create a framework in which the determinants of health are placed in a spatial context. This provides the discipline of health geography, which is rather practice-based, with a theory-based concept. This framework locates factors that affect health and their interrelationships on a spatial scale. While there have been many previous models or conceptual frameworks regarding the determinants of health, thus far they have lacked an overall spatial perspective.

The aforementioned determinants of health and their explanations in Section 2 have shown how diverse and complex the interrelationships are for health. Following Tobler’s first law of Geography that “everything is related to everything else, but near things are more related than distant things” [96], it becomes clear that all determinants of health have a spatial dimension. An exception here would be politics and decision-making processes, which cannot always be assigned to a spatial level. Following Tobler, determinants that are spatially near may have a more significant impact on individual health. Determinants on another spatial scale may also have a direct or indirect impact on a person and his or her health status and are more effective the further their spatial extension or effect is. The advantages of looking at health determinants with a spatial perspective are, first, it provides a better means of identifying these determinants or e.g., the origin of a disease. Second, it gives the possibility to apply an intervention in a targeted way in terms of prevention and treatment. To construct the conceptual framework, it was necessary to deal with the spatial scales first that are relevant in the context of health. The superordinate level distinguishes between macro, meso and micro level. The subordinate level distinguishes between global, national, regional, local, neighbourhood and household level. For better understanding, we assigned examples to the scales. With regard to both the selected scales and the examples, it should be mentioned that the scales generally move along a continuum and cannot and should not always be distinguished from one another. The same applies to the assignment of the examples to the scales. For example, it is possible to distinguish between urban districts and urban quarters and to assign a range between regional and neighbourhood. This, however, is not valid in general but is context dependent. Nevertheless, both the scales and the examples can illustrate a spatial differentiation to which determinants of health can be assigned.

In the conceptual framework, the determinants of health are assigned to the spatial scales and operating mechanisms and pathways are highlighted. As always when describing something as complex as the factors influencing a person’s health, it is important to understand that these mechanisms/pathways do not only run vertically from the macro level (e.g., global) to the micro level (e.g., local) but also vice versa (feedback mechanisms) and horizontally between the determinants of one level (e.g., sociodemographic conditions and healthcare). This once again highlights the complexity of this construct, which represents the conceptual framework in a simplified way. It also shows that aspects that are spatially closer to a person have probably a more direct, faster or greater impact.

The conceptual framework presented here has strengths and limitations. Among the strengths, it should be said that this is the first framework to intersect the determinants of health with a spatial perspective. It generates a working basis for future analyses in which spatial aspects of health are addressed. In addition, the conceptual framework provides a better understanding of the spatial effects of different determinants on health and sensitises the reader to the differentiation of spatial scales in this context. Limitations include the fact that a framework always represents a simplification of reality. Here, this is especially true with regard to the mechanisms of action at the different scales, which can be very complex in reality. In this context, we use the word ‘scale’ as a rather methodological way to distinguish larger areas from smaller areas. Scale can however be perceived differently by different populations or can be conceptionalised differently from the purely cartographic perspective [97,98]. In addition, no exact definition and separation of the spatial scales can be made, as this is usually context dependent. Despite this limitation, the framework all in all provides an important conceptual contribution to the spatial perspective on health and can serve as an overview of the vast complexity of multiple determinants of health.

## Data Availability

Data are available on reasonable request from the corresponding author.

## References

[1] Du Prel J.-B., Röhrig B., Weinmayr G. (2010). Was ist Epidemiologie?. Krankenh. Up2date.

[2] Friis R.H. (2010). Epidemiology for Public Health Practice.

[3] Graunt J. (1662). Natural and Political Observations Made Upon the Bills of Mortality.

[4] Elliott P., Wartenberg D. (2004). Spatial Epidemiology: Current Approaches and Future Challenges. Environ. Health Perspect..

[5] McLeod K.S. (2000). Our sense of Snow: The myth of John Snow in medical geography. Soc. Sci. Med..

[6] Rosenberg M.W. (1998). Medical or health geography? Populations, peoples and places. Int. J. Popul. Geogr..

[7] Dummer T.J. (2008). Health geography: Supporting public health policy and planning. CMAJ.

[8] Cummins S., Curtis S., Diez-Roux A.V., Macintyre S. (2007). Understanding and representing ‘place’ in health research: A relational approach. Soc. Sci. Med..

[9] van Merode F. (2009). Judith D. de Jong: “Explaining medical practice variation–Social organization and institutional mechanisms”, Utrecht, 2007. Z. Gesundh. Wiss..

[10] Voigtländer S., Mielck A., Razum O. (2012). Die Bedeutung des kleinräumigen Kontexts für Gesundheit: Entwurf eines Erklärungsmodells. Das Gesundh..

[11] Wennberg J.E. (2011). Time to tackle unwarranted variations in practice. BMJ.

[12] Voigtländer S., Vogt V., Mielck A., Razum O. (2014). Explanatory models concerning the effects of small-area characteristics on individual health. Int. J. Public Health.

[13] National Academies Press (US) (2002). The Future of the Public’s Health in the 21st Century.

[14] Hosseini Shokouh S.M., Arab M., Emamgholipour S., Rashidian A., Montazeri A., Zaboli R. (2017). Conceptual Models of Social Determinants of Health: A Narrative Review. Iran. J. Public Health.

[15] Dahlgren G., Whitehead M. (1991). Policies and Strategies to Promote Social Equity in Health. Background Document to WHO—Strategy Paper for Europe.

[16] Smedley B.D., Syme S.L., Institute of Medicine (US) Committee on Capitalizing on Social Science and Behavioral Research to Improve the Public’s Health (2000). Promoting Health: Intervention Strategies from Social and Behavioral Research.

[17] Zhang X.-X., Liu J.-S., Han L.-F., Xia S., Li S.-Z., Li O.Y., Kassegne K., Li M., Yin K., Hu Q.-Q. (2022). Towards a global One Health index: A potential assessment tool for One Health performance. Infect. Dis. Poverty.

[18] Al-Lawati J.A. (2017). Diabetes Mellitus: A Local and Global Public Health Emergency!. Oman Med. J..

[19] Tran K.B., Lang J.J., Compton K., Xu R., Acheson A.R., Henrikson H.J., Kocarnik J.M., Penberthy L., Aali A., Abbas Q. (2022). The global burden of cancer attributable to risk factors, 2010–2019: A systematic analysis for the Global Burden of Disease Study 2019. Lancet.

[20] Haththotuwa R.N., Wijeyaratne C.N., Senarath U. (2020). Worldwide epidemic of obesity. Obesity and Obstetrics.

[21] Roth G.A., Mensah G.A., Johnson C.O., Addolorato G., Ammirati E., Baddour L.M., Barengo N.C., Beaton A.Z., Benjamin E.J., Benziger C.P. (2020). Global Burden of Cardiovascular Diseases and Risk Factors, 1990–2019: Update From the GBD 2019 Study. J. Am. Coll. Cardiol..

[22] Zhou B., Perel P., Mensah G.A., Ezzati M. (2021). Global epidemiology, health burden and effective interventions for elevated blood pressure and hypertension. Nat. Rev. Cardiol..

[23] Torp Austvoll C., Gallo V., Montag D. (2020). Health impact of the Anthropocene: The complex relationship between gut microbiota, epigenetics, and human health, using obesity as an example. Glob. Health Epidemiol. Genom..

[24] Barouki R., Kogevinas M., Audouze K., Belesova K., Bergman A., Birnbaum L., Boekhold S., Denys S., Desseille C., Drakvik E. (2021). The COVID-19 pandemic and global environmental change: Emerging research needs. Environ. Int..

[25] Calistri P., Decaro N., Lorusso A. (2021). SARS-CoV-2 Pandemic: Not the First, Not the Last. Microorganisms.

[26] Khojasteh D., Davani E., Shamsipour A., Haghani M., Glamore W. (2022). Climate change and COVID-19: Interdisciplinary perspectives from two global crises. Sci. Total Environ..

[27] Afshari M., Ahmadi Teymourlouy A., Asadi-Lari M., Maleki M. (2020). Global Health diplomacy for noncommunicable diseases prevention and control: A systematic review. Glob. Health.

[28] McCartney G., Hearty W., Arnot J., Popham F., Cumbers A., McMaster R. (2019). Impact of Political Economy on Population Health: A Systematic Review of Reviews. Am. J. Public Health.

[29] Link B.G., Phelan J.C. (2002). McKeown and the Idea That Social Conditions Are Fundamental Causes of Disease. Am. J. Public Health.

[30] Engels F. (1958). The Condition of the Working Class in England.

[31] Chadwick E. (1842). Report on the Sanitary Condition of the Labouring Population of Great Britain.

[32] Taylor R., Rieger A. (1985). Medicine as social science: Rudolf Virchow on the typhus epidemic in Upper Silesia. Int. J. Health Serv..

[33] Ciment J. (1999). Life expectancy of Russian men falls to 58. BMJ.

[34] Daniels G.A. Underlying Influences on Health and Mortality Trends in Post-Industrial Regions of Europe. https://123docz.net/document/2461250-underlying-influences-on-health-and-mortality-trends-in-post-industrial-regions-of-europe.htm.

[35] Walsh D., McCartney G., Collins C., Taulbut M., Batty G.D. (2017). History, politics and vulnerability: Explaining excess mortality in Scotland and Glasgow. Public Health.

[36] McCartney G., McMaster R., Popham F., Dundas R., Walsh D. (2022). Is austerity a cause of slower improvements in mortality in high-income countries? A panel analysis. Soc. Sci. Med..

[37] McCartney G., Walsh D., Fenton L., Devine R. Resetting the Course for Population Health: Evidence and Recommendations to Address Stalled Mortality Improvements in Scotland and the Rest of the UK. https://www.gla.ac.uk/media/Media_852696_smxx.pdf.

[38] Walsh D., Dundas R., McCartney G., Gibson M., Seaman R. (2022). Bearing the burden of austerity: How do changing mortality rates in the UK compare between men and women?. J. Epidemiol. Community Health.

[39] Wilkinson R., Marmot M., Wilkinson R., Marmot M. (2003). Social Determinants of Health: The Solid Facts.

[40] World Health Organization (2008). Closing the Gap in a Generation: Health Equity through Action on the Social Determinants of Health.

[41] Marmot M. (2010). Fair Society, Healthy Lives: The Marmot Review.

[42] van den Berg M., van Poppel M., van Kamp I., Andrusaityte S., Balseviciene B., Cirach M., Danileviciute A., Ellis N., Hurst G., Masterson D. (2016). Visiting green space is associated with mental health and vitality: A cross-sectional study in four european cities. Health Place.

[43] Tiwari P., Nair R., Ankinapalli P., Gulati M., Hingorani P., Rao J. (2015). India’s Reluctant Urbanization: Thinking Beyond.

[44] Adam M., Schikowski T., Carsin A.E., Cai Y., Jacquemin B., Sanchez M., Vierkötter A., Marcon A., Keidel D., Sugiri D. (2015). Adult lung function and long-term air pollution exposure. ESCAPE: A multicentre cohort study and meta-analysis. Eur. Respir. J..

[45] Schneider A., Cyrys J., Breitner S., Kraus U., Peter A., Diegmann V., Neunhäuserer L. Quantifizierung von Umweltbedingten Krankheitslasten Aufgrund der Stickstoffdioxid-Exposition in Deutschland. https://www.umweltbundesamt.de/sites/default/files/medien/421/publikationen/abschlussbericht_no2_krankheitslast_final_2018_03_05.pdf.

[46] Krefis A., Albrecht M., Kis A., Jagodzinski A., Augustin M., Augustin J. (2017). Associations of Noise and Socioeconomic and -Demographic Status on Cardiovascular and Respiratory Diseases on Borough Level in a Large German City State. Urban Sci..

[47] van Kempen E., Babisch W. (2012). The quantitative relationship between road traffic noise and hypertension: A meta-analysis. J. Hypertens..

[48] Hermanski A., McClelland J., Pearce-Walker J., Ruiz J., Verhougstraete M. (2022). The effects of blue spaces on mental health and associated biomarkers. Int. J. Ment. Health.

[49] Maas J., Verheij R.A., de Vries S., Spreeuwenberg P., Schellevis F.G., Groenewegen P.P. (2009). Morbidity is related to a green living environment. J. Epidemiol. Community Health.

[50] Jansson A.K., Lubans D.R., Smith J.J., Duncan M.J., Haslam R., Plotnikoff R.C. (2019). A systematic review of outdoor gym use: Current evidence and future directions. J. Sci. Med. Sport.

[51] Yen H.-Y., Chiu H.-L., Huang H.-Y. (2021). Green and blue physical activity for quality of life: A systematic review and meta-analysis of randomized control trials. Landsc. Urban Plan..

[52] Walker R.E., Keane C.R., Burke J.G. (2010). Disparities and access to healthy food in the United States: A review of food deserts literature. Health Place.

[53] Lebel A., Kestens Y., Pampalon R., Thériault M., Daniel M., Subramanian S.V. (2012). Local context influence, activity space, and foodscape exposure in two canadian metropolitan settings: Is daily mobility exposure associated with overweight?. J. Obes..

[54] Kaczynski A.T., Eberth J.M., Stowe E.W., Wende M.E., Liese A.D., McLain A.C., Breneman C.B., Josey M.J. (2020). Development of a national childhood obesogenic environment index in the United States: Differences by region and rurality. Int. J. Behav. Nutr. Phys. Act..

[55] Lakes T., Burkart K. (2016). Childhood overweight in Berlin: Intra-urban differences and underlying influencing factors. Int. J. Health Geogr..

[56] Penchansky R., Thomas J.W. (1981). The concept of access: Definition and relationship to consumer satisfaction. Med. Care.

[57] Peters D.H., Garg A., Bloom G., Walker D.G., Brieger W.R., Rahman M.H. (2008). Poverty and access to health care in developing countries. Ann. N. Y. Acad. Sci..

[58] Saurman E. (2016). Improving access: Modifying Penchansky and Thomas’s Theory of Access. J. Health Serv. Res. Policy.

[59] Alt K.W., Al-Ahmad A., Woelber J.P. (2022). Nutrition and Health in Human Evolution-Past to Present. Nutrients.

[60] Brooks-Russell A., Simons-Morton B., Haynie D., Farhat T., Wang J. (2014). Longitudinal Relationship Between Drinking with Peers, Descriptive Norms, and Adolescent Alcohol Use. Prev. Sci..

[61] LaBrie J.W., Atkins D.C., Neighbors C., Mirza T., Larimer M.E. (2012). Ethnicity Specific Norms and Alcohol Consumption Among Hispanic/Latino/a and Caucasian Students. Addict. Behav..

[62] Sudhinaraset M., Wigglesworth C., Takeuchi D.T. (2016). Social and Cultural Contexts of Alcohol Use: Influences in a Social–Ecological Framework. Alcohol Res..

[63] Ahern J., Galea S., Hubbard A., Midanik L., Syme S.L. (2008). “Culture of drinking” and individual problems with alcohol use. Am. J. Epidemiol..

[64] Mohammadnezhad M., Tsourtos G., Wilson C., Ratcliffe J., Ward P. (2015). Understanding Socio-cultural Influences on Smoking among Older Greek-Australian Smokers Aged 50 and over: Facilitators or Barriers? A Qualitative Study. Int. J. Environ. Res. Public Health.

[65] Lee H.Y., Im H. (2013). Colorectal cancer screening among Korean American immigrants: Unraveling the influence of culture. J. Health Care Poor Underserved.

[66] Gorin S.S. (2005). Correlates of colorectal cancer screening compliance among urban Hispanics. J. Behav. Med..

[67] Bastani R., Gallardo N.V., Maxwell A.E. (2001). Barriers to Colorectal Cancer Screening Among Ethnically Diverse High-and Average-Risk Individuals. J. Psychosoc. Oncol..

[68] Niedhammer I., Chastang J.-F., David S., Kelleher C. (2008). The contribution of occupational factors to social inequalities in health: Findings from the national French SUMER survey. Soc. Sci. Med..

[69] Kristensen T.S., Borg V., Hannerz H. (2002). Socioeconomic status and psychosocial work environment: Results from a Danish national study. Scand. J. Public Health Suppl..

[70] Hämmig O., Bauer G.F. (2013). The social gradient in work and health: A cross-sectional study exploring the relationship between working conditions and health inequalities. BMC Public Health.

[71] Borg V., Kristensen T.S. (2000). Social class and self-rated health: Can the gradient be explained by differences in life style or work environment?. Soc. Sci. Med..

[72] Lahelma E., Laaksonen M., Aittomäki A. (2009). Occupational class inequalities in health across employment sectors: The contribution of working conditions. Int. Arch. Occup. Environ. Health.

[73] Kaikkonen R., Rahkonen O., Lallukka T., Lahelma E. (2009). Physical and psychosocial working conditions as explanations for occupational class inequalities in self-rated health. Eur. J. Public Health.

[74] Rodrigue J.-P., Comtois C., Slack B. (2006). The Geography of Transport Systems.

[75] Sternlieb F., Laituri M., Brebbia C.A. (2009). Tracking political climate change: US policy and the human right to water. Water Resources Management V.

[76] Amin A.T. Urban Strategy for Sustainable Urbanization in Asian Countries: Some Policy Insights from Sub Regional Comparison. Proceedings of the 17th Conference of the International Association of Historians of Asia.

[77] Meade M.S., Emch M. (2010). Medical Geography.

[78] Helldén D., Andersson C., Nilsson M., Ebi K.L., Friberg P., Alfvén T. (2021). Climate change and child health: A scoping review and an expanded conceptual framework. Lancet Planet. Health.

[79] Bourke L., Humphreys J.S., Wakerman J., Taylor J. (2012). Understanding rural and remote health: A framework for analysis in Australia. Health Place.

[80] World Health Organization European Action Plan for Strengthening Public Health Capacities and Services. https://apps.who.int/iris/bitstream/handle/10665/336410/62wd12e-rev1-EAPPublicHealth-121828.pdf?sequence=1&isAllowed=y.

[81] World Health Organization (1986). Ottawa Charter for Health Promotion. https://www.euro.who.int/__data/assets/pdf_file/0004/129532/Ottawa_Charter.pdf.

[82] Macintyre S. (2007). Inequalities in Health in Scotland: What Are They and What Can We Do about Them.

[83] Kickbusch I., Brindley C., Williams C., Valentine N. (2015). Health in All Policies: Training Manual.

[84] Beeston C., McCartney G., Ford J., Wimbush E., Beck S., MacDonald W., Fraser A. Health Inequalities Policy Review for the Scottish Ministerial Task Force on Health Inequalities. https://www.healthscotland.scot/media/1053/1-healthinequalitiespolicyreview.pdf.

[85] Reintjes R. (2012). Variation matters: Epidemiological surveillance in Europe. J. Health Polit. Policy Law.

[86] Sahal N., Reintjes R., Aro A.R. (2009). Review article: Communicable diseases surveillance lessons learned from developed and developing countries: Literature review. Scand. J. Public Health.

[87] Riggsbee K.A., Riggsbee J., Vilaro M.J., Moret L., Spence M., Steeves E.A., Zhou W., Olfert M.D., Franzen-Castle L., Horacek T. (2019). More than fast food: Development of a story map to compare adolescent perceptions and observations of their food environments and related food behaviors. Int. J. Environ. Res. Public Health.

[88] Thompson C., Ponsford R., Lewis D., Cummins S. (2018). Fast-food, everyday life and health: A qualitative study of ‘chicken shops’ in East London. Appetite.

[89] Kalbus A., de Souza Sampaio V., Boenecke J., Reintjes R. (2021). Exploring the influence of deforestation on dengue fever incidence in the Brazilian Amazonas state. PLoS ONE.

[90] Schweikart J., Kistemann T. (2004). Geoinformationssysteme im Gesundheitswesen: Grundlagen und Anwendungen.

[91] Kim J., Kim D.H., Lee J., Cheon Y., Yoo S. (2022). A scoping review of qualitative geographic information systems in studies addressing health issues. Soc. Sci. Med..

[92] Cohen-Cline H., Turkheimer E., Duncan G.E. (2015). Access to green space, physical activity and mental health: A twin study. J. Epidemiol. Community Health.

[93] Schraufnagel D.E., Balmes J.R., de Matteis S., Hoffman B., Kim W.J., Perez-Padilla R., Rice M., Sood A., Vanker A., Wuebbles D.J. (2019). Health Benefits of Air Pollution Reduction. Ann. Am. Thorac. Soc..

[94] Yiannakoulias N., Svenson L.W., Schopflocher D.P. (2009). An integrated framework for the geographic surveillance of chronic disease. Int. J. Health Geogr..

[95] World Health Organization (2007). Everybody’s Business: Strengthening Health Systems to Improve Health Outcomes: WHO’s Framework for Action.

[96] Tobler W.R. (1970). A Computer Movie Simulating Urban Growth in the Detroit Region. Econ. Geogr..

[97] Oliveira I.J.D., Romão P.D.A. (2021). Geografia e escalas: O lugar das escalas cartográfica, espacial e geográfica. Atelie Geogr..

[98] Csillag F., Fortin M.-J., Dungan J.L. (2000). On the Limits and Extensions of the Definition of Scale. Bull. Ecol. Soc. Am..

